# Integrating predicted transcriptome from multiple tissues improves association detection

**DOI:** 10.1371/journal.pgen.1007889

**Published:** 2019-01-22

**Authors:** Alvaro N. Barbeira, Milton Pividori, Jiamao Zheng, Heather E. Wheeler, Dan L. Nicolae, Hae Kyung Im

**Affiliations:** 1 Section of Genetic Medicine, The University of Chicago, Chicago, Illinois, United States of America; 2 Department of Biology, Loyola University Chicago, Chicago, Illinois, United States of America; 3 Department of Computer Science, Loyola University Chicago, Chicago, Illinois, United States of America; 4 Department of Statistics, The University of Chicago, Chicago, Illinois, United States of America; 5 Department of Human Genetics, The University of Chicago, Chicago, Illinois, United States of America; University College London, UNITED KINGDOM

## Abstract

Integration of genome-wide association studies (GWAS) and expression quantitative trait loci (eQTL) studies is needed to improve our understanding of the biological mechanisms underlying GWAS hits, and our ability to identify therapeutic targets. Gene-level association methods such as PrediXcan can prioritize candidate targets. However, limited eQTL sample sizes and absence of relevant developmental and disease context restrict our ability to detect associations. Here we propose an efficient statistical method (MultiXcan) that leverages the substantial sharing of eQTLs across tissues and contexts to improve our ability to identify potential target genes. MultiXcan integrates evidence across multiple panels using multivariate regression, which naturally takes into account the correlation structure. We apply our method to simulated and real traits from the UK Biobank and show that, in realistic settings, we can detect a larger set of significantly associated genes than using each panel separately. To improve applicability, we developed a summary result-based extension called S-MultiXcan, which we show yields highly concordant results with the individual level version when LD is well matched. Our multivariate model-based approach allowed us to use the individual level results as a gold standard to calibrate and develop a robust implementation of the summary-based extension. Results from our analysis as well as software and necessary resources to apply our method are publicly available.

## Author summary

We develop a new method, MultiXcan, to test the mediating role of gene expression variation on complex traits, integrating information available across multiple tissue studies. We show this approach has higher power than traditional single-tissue methods. We extend this method to use only summary-statistics from public GWAS. We apply these methods to 222 complex traits available in the UK Biobank cohort, and 109 complex traits from public GWAS and discuss the findings.

## Introduction

Recent technological advances allow interrogation of the genome to a high level of coverage and precision, enabling experimental studies that query the effect of genotype on both complex and molecular traits. Among these, GWAS have successfully associated genetic loci to human complex traits. GWAS meta-analyses with ever increasing sample sizes allow the detection of associated variants with smaller effect sizes [1–3]. However, understanding the mechanism underlying these associations remains a challenging problem.

Another approach is the study of expression quantitative trait loci (eQTLs), measuring association between genotype and gene expression. These studies provide a wealth of biological information but tend to have smaller sample sizes. A similar observation applies to QTL studies of other traits such methylation, metabolites, or protein levels.

The importance of gene expression regulation in complex traits [4–7] has motivated the development of methods to integrate eQTL studies and GWAS. To examine these mechanisms we developed PrediXcan [8], which tests the mediating role of gene expression variation in complex traits. Briefly, PrediXcan tests the hypothesis that genetic variants affect phenotypes through the regulation of gene expression traits. To do that, it correlates genetically predicted gene expression and the phenotype with the idea that causal genes are likely to show a significant association. Linear prediction models of expression using genetic variation in the vicinity of the gene are trained in reference transcriptome datasets such as Genotype-Tissue Expression project (GTEx) [9].

Due to sharing of eQTLs across multiple tissues, we have shown the benefits of an agnostic scanning across all available tissues [10]. Despite the increased multiple testing burden (for Bonferroni correction, the total number of gene-tissue pairs must be used when determining the threshold), we gain considerably in number of significant genes. However, given the substantial correlation between different tissues [9], Bonferroni correction can be too stringent increasing the false negative rate.

In order to aggregate evidence more efficiently, we present here a method termed MultiXcan, which tests the joint effects of gene expression variation from different tissues. Furthermore, we develop and implement a method that only needs summary statistics from a GWAS: Summary-MultiXcan (S-MultiXcan). We make our implementation publicly available to the research community in https://github.com/hakyimlab/MetaXcan. We apply this method to simulated and real data (222 traits from the UK Biobank study [11] and 109 public GWAS) to show the performance and proper calibration of p-values. We make all of the results publicly available at https://doi.org/10.5281/zenodo.1402225.

## Results

### MultiXcan combines information across tissues using multivariate regression

To integrate information across tissues, MultiXcan regresses the phenotype of interest on the predicted expression of a gene in multiple tissues as follows:

$$\begin{array}{c} y=\mu +t_{1}g_{1}+t_{2}g_{2}+\cdots +t_{p}g_{p}+e \end{array}$$ (1)

where **y** is the *n*-dimensional phenotype vector, ***μ*** is an intercept term, **t**_*i*_ is standardized predicted expression of the gene in tissue *i*, *g*_*i*_ is its effect size, and **e** an error term with variance $\sigma_{e}^{2}$; *p* is the number of available tissue models. We use an *F*-test to assess the joint significance of the regression.

**Fig 1:**
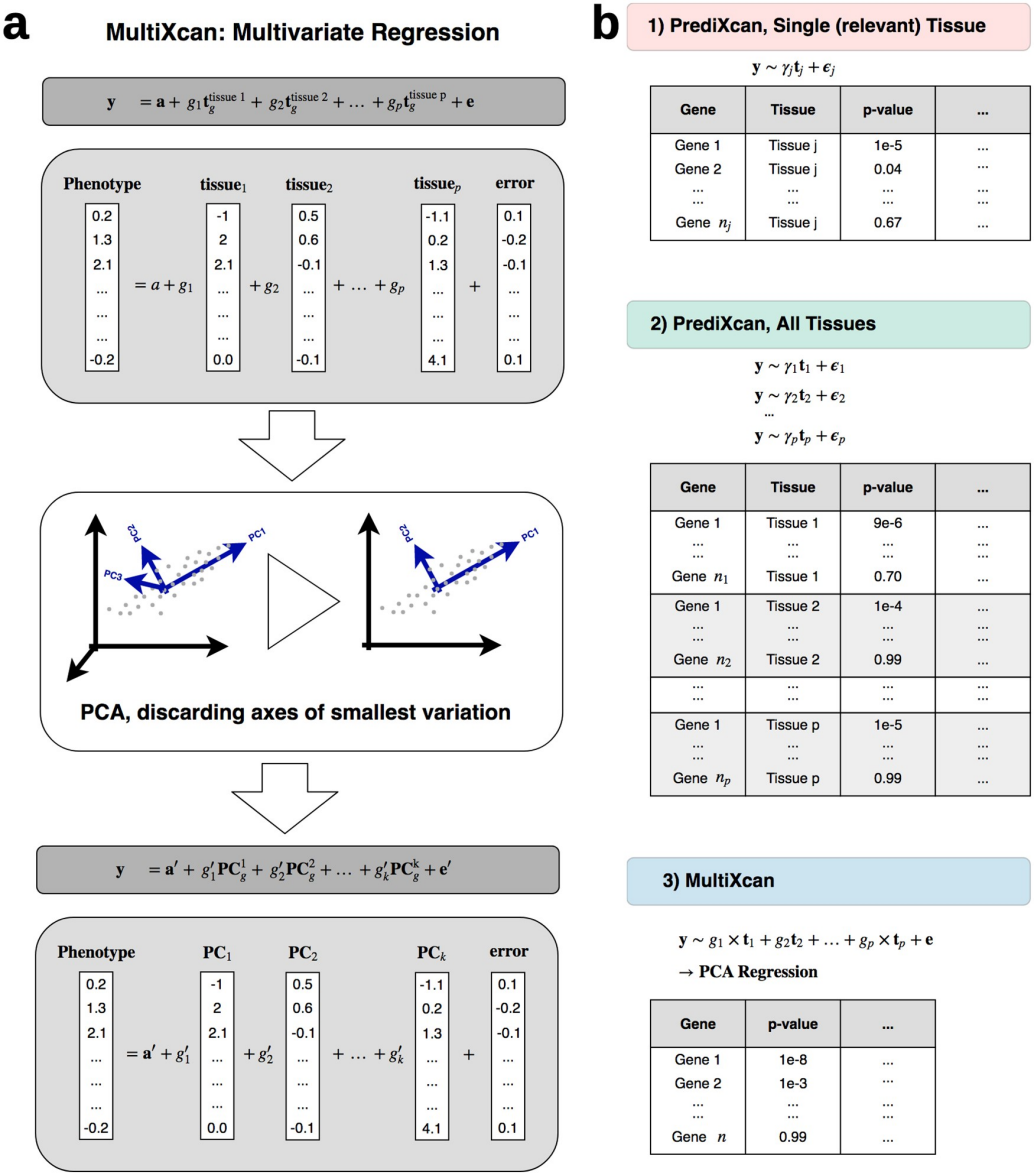
MultiXcan method. **Panel a** illustrates the MultiXcan method. Predicted expression from all available tissue models are used as explanatory variables. To avoid multicolinearity, we use the first k Principal Components of the predicted expression. **y** is a vector of phenotypes for *n* individuals, $t_{g}^{\text{tissue}\;j}$ is the standardized predicted gene expression for tissue *j*, **g**_*j*_ is its effect size, **a** is an intercept and **e** is an error term. **Panel b** shows a schematic representation of MultiXcan results compared to classical PrediXcan, both for a single relevant tissue and all available tissues in agnostic scanning. **y** is a (centered) vector of phenotypes for *n* individuals, **t**_*j*_ is the standardized predicted gene expression for model *j*, *g*_*j*_ is its effect size in the joint regression, *γ*_*j*_ is its effect size in the marginal regression using only prediction *j*, **e** and *ϵ*_*j*_ are error terms.

Expression predictions across tissues can be highly correlated. We predicted expression for individuals from the UK Biobank cohort using models trained on 44 GTEx tissues (as presented in [10]), and found a median pair-wise correlation of *r*_*p*50_ = 0.56 (*IQR* = 0.69) between different tissue models in a given gene, across genes (see Methods for details). To avoid numerical issues caused by collinearity, we use principal components of the predicted expression data matrix as explanatory variables, and discard the axes of smallest variation (PCA regularization). Additional covariates can be added to the regression seamlessly. Fig 1-a displays an overview of the method; see further details in the Methods section. S1 Fig shows an example of the correlation between tissues of predicted expression of the gene *SLC5A6*.

### MultiXcan detects more associations than single-tissue PrediXcan

We applied MultiXcan to 222 traits from the UK Biobank cohort. The traits were chosen based on several criteria, such as availability of well-established literature, binary traits having enough cases, or potential interest for a phenome-wide study (allergy, behavioral, metabolic and anthropometric phenotypes). We used Elastic Net prediction models trained on 44 tissues from GTEx, originally presented in [10].

We compared three approaches for assessing the significance of a gene jointly across all tissues: 1) running PrediXcan using the most relevant tissue; 2) running PrediXcan using all tissues, one tissue at a time; 3) running MultiXcan. Fig 1-b illustrates the results from each approach. We summarize a comparison between approaches 2) and 3) in Table 1. PrediXcan overcomes MultiXcan only in 21 traits, all of them with less than 50 significant associations across both methods. MultiXcan detects more associations in 103 traits.

**Table 1:** Summary statistics comparing MultiXcan and PrediXcan on UK Biobank.

Traits with more MultiXcan-significant associations	103
Traits with more PrediXcan-significant associations	21
Tied traits	6
Traits without significant associations	92
Average increase in significant associations for MultiXcan *	162.7
Average significant association overlap **	48.0%

*: average performed across traits where there is at least one PrediXcan- or MultiXcan-significant association.

**: computed as $\frac{\#\text{shared}}{\#union(M,P)}$, with *M* the MultiXcan-significant associations, *P* the PrediXcan-significant associations, and #shared the number of shared associations.

**Fig 2:**
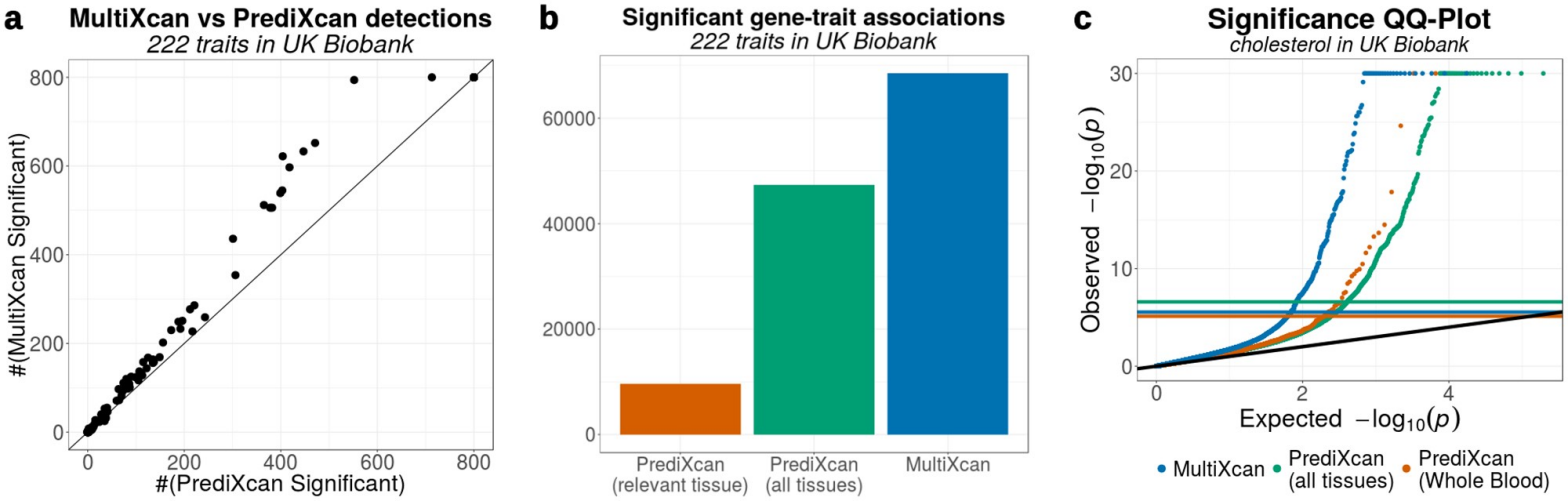
Improved significance of MultiXcan vs PrediXcan across a broad set of traits. **Panel a** compares the number of significant associations detected by MultiXcan and PrediXcan for 222 traits from UK Biobank. These numbers were thresholded at 800 for visualization purposes. **Panel b** shows the number of discoveries in each method across the 222 UK Biobank traits. MultiXcan is able to detect more findings PrediXcan, either with a single tissue or using all 44 GTEx tissues. **Panel c** compares the distribution of MultiXcan’s p-values to PrediXcan’s p-values for the Cholesterol trait in the UK Biobank cohort. Both PrediXcan with a single tissue model (GTEx Whole Blood) and 44 models (GTEx v6p models) are shown. Notice that Bonferroni-significance levels are different for each case, since 6588 genes were tested in PrediXcan for Whole Blood, 195532 gene-tissue pairs for all GTEx tissues, and 17434 genes in MultiXcan. P-values were truncated at 10^−30^ for visualization convenience.

Fig 2-a and 2-b show a comparison of detections for both MultiXcan and PrediXcan. See S1 Dataset for a summary of detections per trait, and S2 and S3 Datasets for the full list of significant MultiXcan and PrediXcan results respectively.

As an illustrative example, we examined more closely the results for self-reported high cholesterol phenotype (http://biobank.ctsu.ox.ac.uk/crystal/field.cgi?id=20002). We used 50,497 cases and 100,994 controls. After Bonferroni correction, MultiXcan was able to detect a larger number of significantly associated genes (251 detections) than PrediXcan using all tissues (196 detections) or only a single tissue (whole blood, 33 detections). 172 genes were detected by both PrediXcan and MultiXcan. Fig 2-c shows the QQ-plot for associations in these three approaches. There are 79 genes associated to high cholesterol via MultiXcan and not PrediXcan. Among them, we find genes related to lipid metabolism (*APOM* [12], *PAFAH1B2* [13]), glucose transport(*SLC5A6* [14]), and vascular processes (*NOTCH4* [15]). The well known gene *SORT1* is detected by both MultiXcan and PrediXcan.

### Performance and calibration of MultiXcan in simulated traits

To evaluate MultiXcan’s performance in different known scenarios, we simulated traits as a function of different numbers of causal tissues for each gene: a single tissue, multiple tissues, all available tissues. We executed PrediXcan, MultiXcan without PCA regularization, and MultiXcan with PCA regularization. We show proper calibration under the null hypothesis of no association in S3 Fig, and robustness of the regularization approach in S6 Fig. See further details in S1 Supplementary Note.

As expected, when there is a known single causal tissue, PrediXcan with the known tissue yields more significant associations. However, when there are multiple causal tissues, MultiXcan yields more significant associations than the best single tissue PrediXcan results. In traits simulated from a single causal tissue, PrediXcan outperforms MultiXcan in 99.9% of the cases (AOV p-value < 10^−16^). MultiXcan performs best in scenarios with multiple causal tissues (84.4% of the times when a few tissues are causal, and 99.5% when all tissues are causal; AOV p-value < 10^−16^ in both cases).

One caveat is that the simulation does not cover cases when the prediction in the single tissue has low quality. In such an scenario, borrowing information from other tissues will still be beneficial.

### MultiXcan results can be inferred from GWAS summary results

To expand the applicability of our method to massive sample sizes and to studies where individual level data are not available, we extend our method to use summary results rather than individual-level data. We call this extension Summary-MultiXcan (S-MultiXcan).

**Fig 3:**
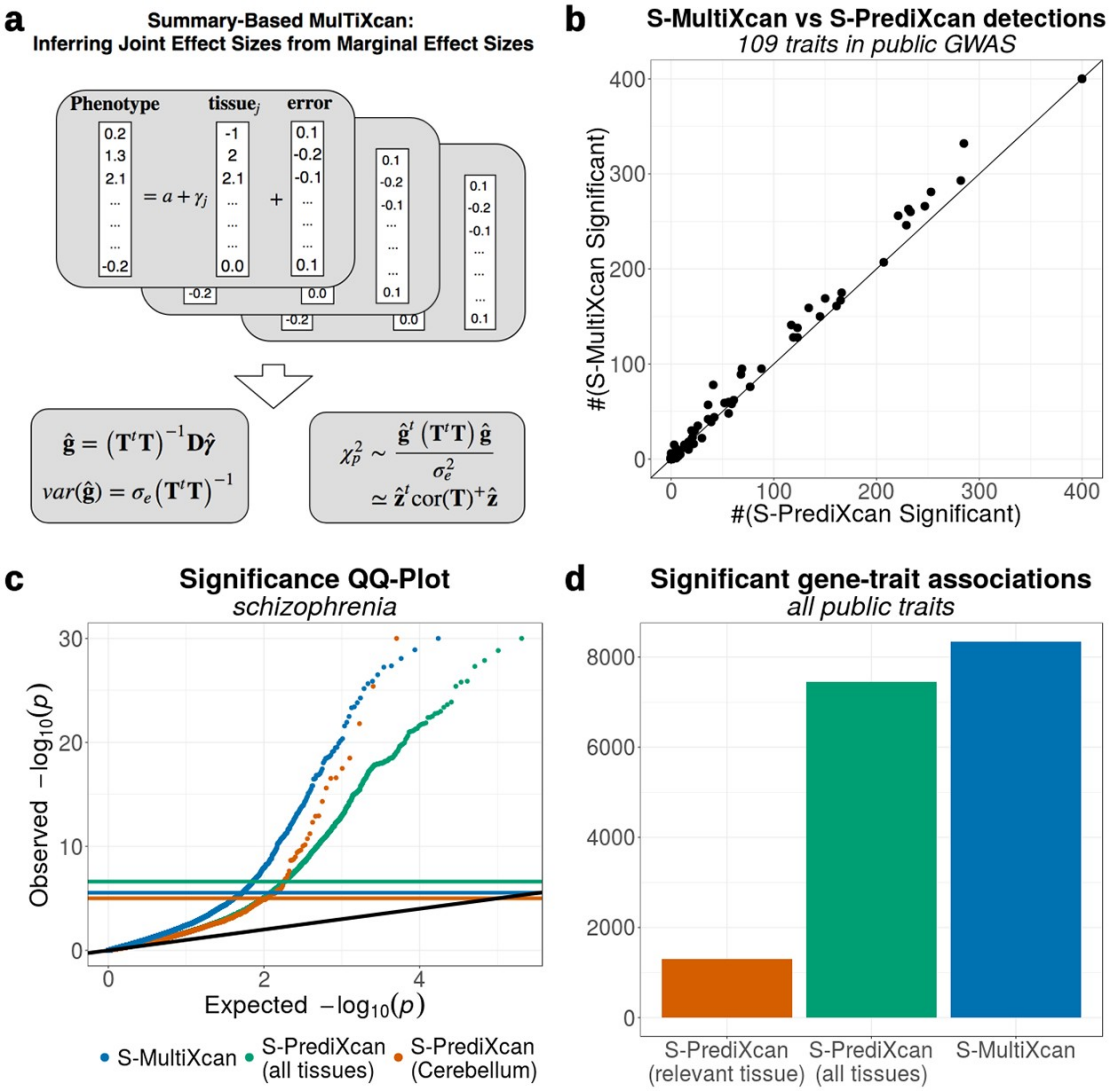
MultiXcan results can be inferred from GWAS summary statistics and a reference panel. **Panel a** illustrates the S-MultiXcan method: the joint effect sizes are inferred from the marginal univariate effect sizes obtained from S-PrediXcan. Significance is quantified using the estimated covariance of the multivariate effect sizes. With the approximations described in Methods, the final *χ*^2^ statistics ends up being equivalent to the omnibus test. **Panel b** compares the number of associations significant via S-MultiXcan versus those significant via S-PrediXcan, for the same GWAS Studies. In most cases, S-MultiXcan detects a larger number of significant associations. The number of discoveries was thresholded at 200 for visualization purposes. **Panel c** displays QQ-Plots for the association p-values from S-MultiXcan and S-PrediXcan in Schizophrenia, using a model trained on brain’s cerebellum, and S-PrediXcan associations for all 44 GTEx tissues. **Panel d** shows the number of significant associations across all public GWAS traits for each method as a bar plot.

We infer the joint estimates of effect sizes of predicted expression on phenotype (Eq 1) using the marginal estimates. We also compute the covariance matrix of the effect sizes and leverage the asymptotic multivariate normality of the estimates, to compute a statistic that is approximately $\chi_{p}^{2}$ (*p* number of tissues). The final expression is equivalent to the omnibus test mentioned in [16], which can be interpreted as a specific case of general weighted association analysis [17]. Fig 3-a illustrates our approach and the details can be found in the Methods section.

As with the individual level approach, the correlation between tissues leads to numerical problems (due to near singular covariance matrices that need to be inverted). We address this by using a pseudo inverse approach which, in a nutshell, uses singular value decomposition (SVD) of the covariance matrix to keep only the components of large variation. This is analogous to the PCA regularization used for the individual level approach. Thus we test for significance using $\chi_{k}^{2}$ with *k* the number of surviving components. See details in the Methods Section.

A robust implementation for calculating predicted expression correlation is critical to avoid unnecessary false positive results. In principle, it is possible to simply calculate the correlation between tissues using predicted expression in a reference set. However, we found that this approach can lead to large differences between the individual level data results (our gold standard) and the summary level ones when SNPs from the reference set are missing in the GWAS results. An example of this is shown in S8 Fig with the Type 1 Diabetes study from the Wellcome Trust Case-Control Consortium (WTCCC); association data is included in S9 Dataset. To avoid this problem, we calculate the covariance matrix between tissues using only the predictor SNPs that are common in both the GWAS summary and the reference LD set.

**Fig 4:**
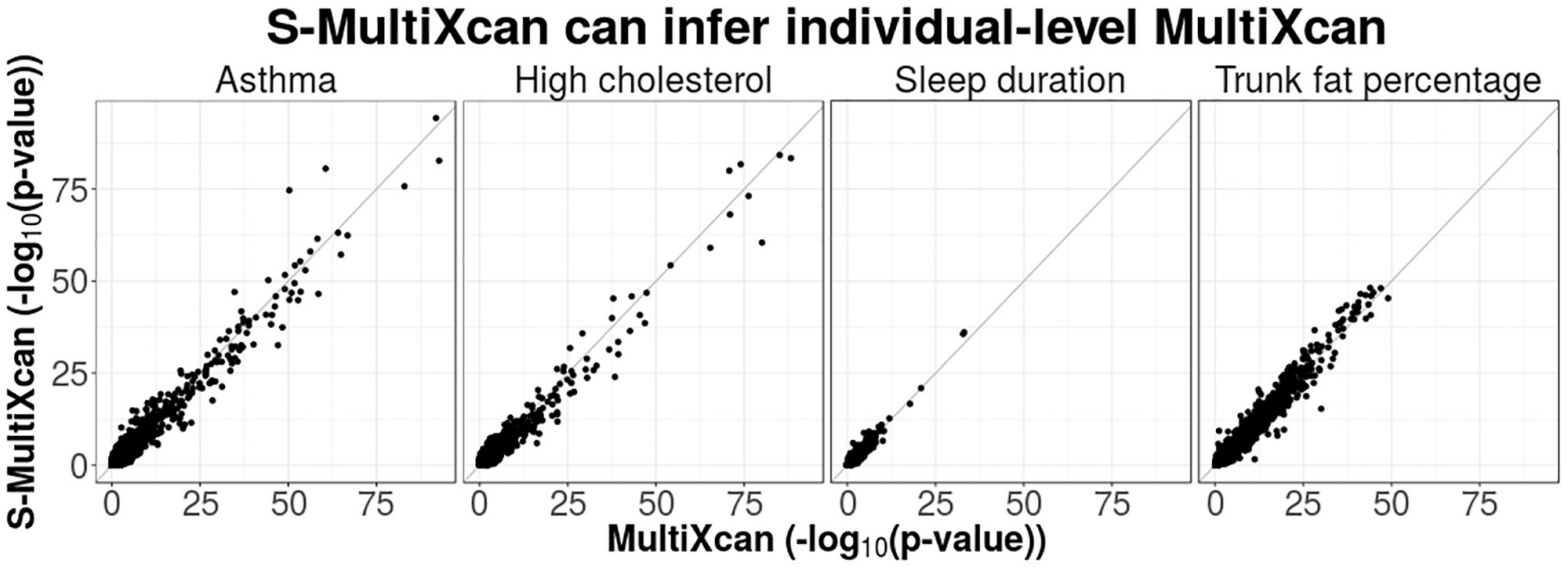
Comparison between S-MultiXcan and individual-level MultiXcan. This figure compares S-MultiXcan to MultiXcan in four UK Biobank phenotypes. GTEx individuals were used as a reference panel for estimating expression correlation in the study population. The summary data-based method shows a good level of agreement with the individual-based method. In cases where the LD-structure between reference and study cohorts is mismatched, the summary-based method becomes less accurate. For example in Asthma, two genes are overestimated; however it tends to be conservative for most genes.

Fig 4 displays a few examples of the general agreement between the individual-level MultiXcan and S-MultiXcan. The summary-based version’s results tend to be slightly more conservative than MultiXcan, as illustrated in S2 Fig. As a general comparison to the individual-level method, we list a summary of S-MultiXcan’s application to the 222 UK Biobank traits on Table 2; we observe an adequate similarity between S-MultiXcan’s and MultiXcan’s summaries. The small loss in power arises from the imperfect match of LD between the UK cohort and the reference panel.

**Table 2:** Summary statistics comparing S-MultiXcan and S-PrediXcan on UK Biobank.

Traits with more S-MultiXcan-significant associations	102
Traits with more S-PrediXcan-significant associations	22
Tied traits	14
Traits without significant associations	84
Average increase in significant associations for S-MultiXcan *	125.5
Average significant association overlap **	50.0%

*: average performed across traits where there is at least one PrediXcan- or MultiXcan-significant association.

**: computed as $\frac{\#\text{shared}}{\#union(SM,SP)}$, with *SM* the S-MultiXcan-significant associations, *SP* the S-PrediXcan-significant associations, and #shared the number of shared associations.

To reduce false positives due to LD misspecification when dealing with GWAS summary statistics, we discard any significant association result for a gene if the best single tissue result has p-value greater than 10^−4^ (“suspicious associations”). In other words, we keep significant associations if at least one single gene-tissue pair association is borderline significant or better (10^−5^ is the Bonferroni threshold for a typical tissue model). This is rather conservative since it is possible that evidence with modest significance from weakly correlated tissues can lead to very significant combined association when their effects get aggregated. For example among Bonferroni significant genes in the individual level analysis, a median of 8.3% across traits (IQR = 5.7%) have the most significant marginal (PrediXcan) p-value greater than 10^−4^. We list the number of such genes for each of the 222 UK Biobank traits in S8 Dataset.

### Application to a broad set of complex traits with only summary results

We applied S-MultiXcan to 109 traits on publicly available GWAS, chosen with a similar criteria as UK Biobank’s traits. Like the individual level method, we observed S-MultiXcan to detect more associations than S-PrediXcan in most cases (average detection increase 10), as shown in Fig 3-b, after discarding suspicious associations. We also show the QQ-plots for a sample trait (Schizophrenia) on Fig 3-c and the total number of associations across all public GWAS traits in 3-d.

We display a summarized comparison between S-MultiXcan and S-PrediXcan in S1 Table, after discarding suspicious associations. The list of analyzed traits can be found in S4 and S5 Datasets contains a summary of significant associations for each trait and for each method. S6 Dataset lists the significant S-MultiXcan results for each trait. These results have been uploaded to https://doi.org/10.5281/zenodo.1402225.

#### New associations identified by S-MultiXcan

We examine below the biological relevance of a few of the genes detected by our new method that was missed when using one tissue at a time (S-PrediXcan).

For example, in the Early Growth Genetics (EGG) Consortium’s Body-Mass Index (BMI) study, S-MultiXcan detects three genes not significant in S-PrediXcan: *POMC* (p-value = 1.4 × 10^−6^, tied to childhood obesity [18]); *RACGAP1* (p-value = 1.2 × 10^−10^; embryogenesis [19], cell growth and differentiation, [20]); and *TUBA1B* (p-value = 1.23 × 10^−09^, circadian cycle processes and psychological disorders [21], suggesting a behavioral pathway).

In the CARDIoGRAM+C4D Coronary Artery Disease (CAD) study, S-MultiXcan detected 12 associations not significant in S-PrediXcan. The top result was *AS3MT* (p-value = 4.3 × 10^−9^), related to arsenic metabolism; interestingly, environmental and toxicological studies link arsenic exposure and *AS3MT* polymorphisms with cardiovascular disease [22, 23]. Associations previously linked to CAD included *CDKN2B* (p-value<1.0 × 10^−6^, [24]) *HECTD4* (p-value<2.3 × 10^−6^, [25]). Other interesting S-MultiXcan findings were *CLCC1* (pvalue = 1.2 × 10^−7^, a gene for chloride channel activity); *IREB2* (p-value = 2.1 × 10^−7^, recently linked to pulmonary conditions, [26]), and *ADAM15* (p-value = 2.5 × 10^−07^, from the disintegrin and metalloproteinase family, linked to atherosclerosis [27], atrial fibrillation [28], and other vascular processes [29, 30]).

The list of significant S-MultiXcan and S-PrediXcan results for all traits can be found in S6 and S7 Datasets.

## Discussion

Motivated by the widespread sharing of regulatory processes across tissues [9], we propose MultiXcan, a method that aggregates information by jointly fitting the phenotype on predicted expression across multiple tissues. In simulations and real data, we show that our approach can detect more associations. To expand the applicability of our approach, we derive the analytical expression to infer the association using summary results only, which we show is approximately equivalent to the omnibus test. An important benefit of our multivariate approach is that we can use the individual level data as gold standard to calibrate the type and degree of regularization needed to invert the near singular covariance matrices found in practice. The availability of a gold standard also allowed to identify the need for robust estimates of correlations between tissues.

We found high concordance, in general, between the individual level and summary version with the latter slightly more conservative. As any method relying on a reference panel, S-MultiXcan may be inaccurate when the study population has a different LD structure than the reference panel. We attempted to address this by flagging results where none of the marginal associations reached a somewhat arbitrary threshold of 10^−4^. This is far from perfect. To take full advantage of summary results and summary-based methods, reference sets that are the closest to the study population should be used. This also stresses the need to generate representative reference LD datasets for a wide variety of populations.

Via simulations, we show that MultiXcan is properly calibrated under the null hypothesis of no associations. This is reassuring, but it is possible that in real data there are hidden confounders that we did not capture in our simulations. For example, significant association results might arise due to LD contamination, i.e. when causal variants for the trait and expression are different but in LD with each other, inducing a spurious correlation between the predicted expression and the trait. This is a complex problem that we are currently working to address. In Barbeira et al [10], we sought to address the LD contamination issue by adding a colocalization filtering step where we discard associations with low colocalization probability, using COLOC [31] to keep only associations with *P*_colocalized_ > 0.5. A similar strategy may be applied for MultiXcan by restricting the analysis to gene-tissue pairs with high colocalization probability in the marginal analysis.

In practice, we emphasize the need to further validate the significant associations with additional replication and experimental follow-up.

Importantly, we provide compelling examples where using multiple tissues rather than picking one considered to be relevant for the phenotype increases the list of candidate causal genes. In our simulations, we found that only when the single causal tissue is known and the regulatory mechanism is captured perfectly by predicted expression in that tissue, using PrediXcan with that tissue yields more significant associations than MultiXcan. This scenario is unlikely to occur in practice. Therefore, in general, we recommend jointly scanning of all tissues in addition to focusing on a few tissues selected based on prior knowledge.

### Software and resources

We make our software publicly available on a GitHub repository: https://github.com/hakyimlab/MetaXcan. Prediction model weights and covariances for different tissues can be downloaded from http://predictdb.org/. A short working example can be found on the GitHub page; more extensive documentation can be found on the project’s https://github.com/hakyimlab/MetaXcan/wiki. The results of S-MultiXcan applied to the 44 human tissues and a broad set of phenotypes can be queried on http://gene2pheno.org. The data used in this paper is publicly available in https://doi.org/10.5281/zenodo.1402225.

## Materials and Methods

### Ethics statement

This study uses de-identified genotype and phenotype data from public repositories including dbGaP, EGA, and UK Biobank. Our study has been determined to be non-human subject research by the University of Chicago’s IRB protocol number IRB16-0921.

#### Definitions, notation and preliminaries

In the following, we shall denote scalar quantities by italicized lower-case letter (e.g. *a*); vector quantities with bold lower-case letters (e.g., **a**) and matrices with bold capital letters (e.g. **A**). Corresponding scalar entries will be denoted by subscripts (e.g. *a*_*i*_ is the *i*-th entry for vector **a**).

Let us consider a GWAS study of *n* samples, and assume availability of prediction models in *p* different tissues. Each model *j* is a collection of prediction weights $w_{i}^{j}$.

Let:

- **y** be an *n*-vector of phenotypes, assumed to be centered for convenience.
- **X** the genotype matrix, where each column *X*_*l*_ is the *n*-vector genotype for SNP *l*. We assume it coded in the range [0, 2] but it can be defined in another range, or standardized.
- ${\tilde{t}}_{j}=\sum_{i\in {\text{model}}_{j}}w_{i}^{j}X_{i}$ be the predicted expression in tissue *j*. This is the independent variable used by single-tissue PrediXcan. A prediction model *j* is defined by the set of weights $\left\{w_{i}^{j}\right\}$.
- **t**_*j*_ be the standardization of ${\tilde{t}}_{j}$ to *mean* = 0 and *standard deviation* = 1.

In our application, different genes have different numbers of available tissue models trained on GTEx data, ranging up to *p* = 44. This method is easily extensible to support incorporation of other covariates, or correction by them.

### MultiXcan

MultiXcan consists of fitting a linear regression of the phenotype on predicted expression from multiple tissue models jointly:

$$\begin{array}{cc} y & =\sum_{j=1}^{p}t_{j}g_{j}+e\\ & =Tg+e \end{array}$$ (2)

where **y** is a centered vector of phenotypes for *n* individuals, **t**_*j*_ is an *n*-vector of standardized predicted gene expression for model *j*, *g*_*j*_ is the effect size for the predicted gene expression *j*, **e** is an error term with variance $\sigma_{e}^{2}$, and *p* is the number of tissues. Thus, **T** is a data matrix where each column *j* contains the values from **t**_*j*_, and **g** is the *p*-vector of effect sizes *g*_*j*_.

The high degree of eQTL sharing between different tissues induces a high correlation between predicted expression levels. In order to avoid collinearity issues and numerical instability, we decompose the predicted expression matrix into principal components and keep only the eigenvectors of non negligible variance. To select the number of components, we used a condition number threshold of $\frac{\lambda_{\text{max}}}{\lambda_{i}}<30$, where *λ*_*i*_ is an eigenvalue of the matrix **T**^*t*^ **T**. As a side effect, we observe moderate increases in significance levels because less informative components of tissue expression are discarded from the model. A range of values between 10 and 100 yielded similar results in the simulations described in S1 Supplementary Note as displayed in S6 Fig.

Lastly, we use an F-test to quantify the significance of the joint fit.

We use Bonferroni correction to determine the significance threshold. For MultiXcan, we use the total number of genes with a prediction model in at least one tissue, which yields a threshold approximately at 0.05/17500 ∼ 2.9 × 10^−6^. For PrediXcan across all tissues, we use the total number of gene-tissue pairs, which yields a threshold approximately at 0.05/200, 000 ∼ 2.5 × 10^−7^. Since the tested hypotheses are not independent, Bonferroni correction is overly conservative, as can be seen when counting the number of associations via FDR in S7 Fig.

#### Application to UK Biobank data

UK Biobank genotype data for 487, 409 individuals was downloaded and processed in the Bionimbus Protected Data Cloud (PDC https://bionimbus-pdc.opensciencedatacloud.org/), a secure biomedical cloud operated at FISMA moderate as IaaS with an NIH Trusted Partner status for analyzing and sharing protected datasets. We computed GWAS results using BGENIE, a program for efficient GWAS for multiple continuous traits [32]. We selected 222 traits available for these individuals, covering continuous phenotypes such as height and self reported diseases such as asthma, prioritizing potential interest for a phenome-wide study (allergy, behavioral, metabolic, anthropometric and common disease phenotypes) and literature availability. We used different covariate groups for these phenotypes as in [33].

We computed gene expression on all individuals using 44 models trained on GTEx release v6p (presented in [10]). For every gene, we computed correlation between available tissues, and then obtained the median correlation from all tissue pairs across all genes.

To allow for uniform correction of unwanted variation, we treated all traits as quantitative and adjusted for the same covariates reported in [33]. These covariates include the first ten genotype principal components, sex, age, genotyping array, and depending on the trait, others such as body mass index (BMI), weight or height. For diseases, we randomly sampled twice as many healthy controls as there were cases. PrediXcan was computed for all tissue-trait combinations and MultiXcan was computed for all traits. For the MultiXcan-significant associations in the 222 traits, the median number of available models is 11 (IQR = 9), with ∼77% components surviving PCA thresholding.

On most continuous phenotypes, there were between 300, 000 and 400, 000 individuals with available data determined by the intersection of covariates and traits. For the case of self reported diseases, we found a number of cases ranging from a few hundreds (i.e. Acne) to 50, 000 (i.e. High Cholesterol).

### Summary-MultiXcan

We have demonstrated that S-PrediXcan can accurately infer PrediXcan results from GWAS Summary Statistics and LD information from a reference panel [10], with the added benefits of reduced computational and regulatory burden. Here we extend MultiXcan in a similar fashion.

Summary-MultiXcan (S-MultiXcan) infers the individual-level MultiXcan results, using univariate S-PrediXcan results and LD information from a reference panel. It consists of the following steps:

- Computation of single tissue association results with S-PrediXcan.
- Estimation of the correlation matrix of predicted gene expression for the models using the Linkage Disequilibrium (LD) information from a reference panel (typically GTEx or 1000 Genomes [34])
- Discarding components of smallest variation from this correlation matrix to avert collinearity and numerical problems (Singular Value Decomposition, analogue to PC analysis in individual-level data).
- Estimation of joint effects from the univariate (single-tissue) results and expression correlation.
- Discarding suspicious results, suspect to be false positives arising from LD-structure mismatch.

#### Joint analysis estimation from marginal effects

To derive the multivariate regression (2) effect sizes and variances using the marginal regression (3) estimates, we employ a technique presented in [35].

More specifically, we want to obtain the multivariate regression coefficient estimates for *g*_*j*_ (2) using the estimates from the marginal regression:

$$\begin{array}{c} y=t_{j}\gamma_{j}+\epsilon_{j} \end{array}$$ (3)

where we assume **y** is centered for convenience (so that no intercept term is needed), and *ϵ*_*j*_ is the marginal regression error term with variance $\sigma_{\epsilon }^{2}$ (i.e. we assume a common variance $\sigma_{\epsilon }^{2}$ for all *j*).

First, notice that the solution to the multivariate regression in Eq (2) is

$$\begin{array}{cc} \hat{g} & ={\left(T^{t}T\right)}^{-1\;}T^{t}y \end{array}$$ (4)

$$\begin{array}{cc} \text{var}(\hat{g}) & =\sigma_{e}^{2}{\left(T^{t}T\right)}^{-1} \end{array}$$ (5)

whereas the solution to the marginal regression in Eq (3) is:

$$\begin{array}{cc} \hat{\gamma } & =D^{-1\;}T^{t}y \end{array}$$ (6)

$$\begin{array}{cc} \text{var}(\hat{\gamma }) & =\sigma_{\epsilon }^{2}D^{-1}\;\;\;\text{with}\;D=\text{diag}\left(T^{t}T\right) \end{array}$$ (7)

where *γ* is the vector of effect sizes *γ*_*j*_. Please note that, since the **t**_*j*_ are standardized, then D=(n−1)1 (1 being the *p* × *p* identity matrix) and $se(\gamma_{j})=\sqrt{var(\gamma_{j})}=\frac{\sigma_{\epsilon }}{\sqrt{n-1}}$.

From (6) we get $T^{t}y=D\hat{\gamma }$, which we replace in (4) and obtain the relationship between marginal and joint estimates:

$$\begin{array}{c} \hat{g}={\left(T^{t}T\right)}^{-1\;}D\hat{\gamma } \end{array}$$ (8)

To compute the variance of the estimated effect sizes (5) we use the variance of the phenotype as a conservative estimate of $\sigma_{e}^{2}$ and LD information from reference samples as described next.

#### Estimating expression correlation from a reference panel

As the genotypes from most GWAS are typically unavailable, we must use a reference panel to compute **T**^*t*^ **T**, using only those SNPS available in the GWAS results. To do so, notice that:

$$\begin{array}{cc} \frac{{\left(T^{t}T\right)}_{ij}}{n-1} & =Cor(t_{i},t_{j})\\ & =Cov(t_{i},t_{j})\\ & =\frac{Cov({\tilde{t}}_{i},{\tilde{t}}_{j})}{\sqrt{\hat{\text{var}}\left({\tilde{t}}_{i}\right)\hat{\text{var}}\left({\tilde{t}}_{j}\right)}}\\ & =\frac{Cov(\sum_{a\in {\text{model}}_{i}}w_{a}^{i}X_{a},\sum_{b\in {\text{model}}_{j}}w_{b}^{j}X_{b})}{\sqrt{\hat{\text{var}}\left({\tilde{t}}_{i}\right)\hat{\text{var}}\left({\tilde{t}}_{j}\right)}}\\ & =\frac{\sum_{\begin{array}{c} a\in {\text{model}}_{i}\\ b\in {\text{model}}_{j} \end{array}}w_{a}^{i}w_{b}^{j}Cov(X_{a},X_{b})}{\sqrt{\hat{\text{var}}\left({\tilde{t}}_{i}\right)\hat{\text{var}}\left({\tilde{t}}_{j}\right)}}\\ & =\frac{\sum_{\begin{array}{c} a\in {\text{model}}_{i}\\ b\in {\text{model}}_{j} \end{array}}w_{a}^{i}w_{b}^{j}\Gamma_{ab}}{\sqrt{\hat{\text{var}}\left({\tilde{t}}_{i}\right)\hat{\text{var}}\left({\tilde{t}}_{j}\right)}} \end{array}$$ (9)

where Γ_*ij*_ are the elements of the covariance matrix $\Gamma =\hat{\text{var}}\left(X\right)={\left(X-\bar{X}\right)}^{t}(X-\bar{X})/(n-1)$. We compute the variances as in the S-PrediXcan analysis:

$$\begin{array}{cc} \hat{\text{var}}\left({\tilde{t}}_{j}\right) & =\hat{\sigma_{j}^{2}}\\ & ={\left(W^{j}\right)}^{t}\Gamma^{j}W^{j}\\ & =\underset{\begin{array}{c} a\in {\text{model}}_{j}\\ \begin{array}{c} b\in {\text{model}}_{j} \end{array} \end{array}}{\sum }w_{a}^{j}w_{b}^{j}\Gamma_{ab}^{j} \end{array}$$ (10)

We restrict the computation to using only SNPS in the intersection between reference panel and GWAS. Failing to do so may lead to inaccurate inference of predicted expression covariance, typically underestimating correlation, leading to false positives as can be seen in S8 Fig.

#### Addressing singularity of the correlation matrix

Given the high degree of correlation among many of the prediction models, **T**^*t*^ **T** is often close to singular and its inverse cannot be reliably calculated for many genes. To address this problem, we compute the pseudo-inverse via Singular Value Decomposition, decomposing the correlation matrix into its principal components and removing those with small eigenvalues (SVD regularization). In other terms, we will restrict the analysis to axes of largest variation of the expression data. This is analogous to the principal components-based approach used with individual level data. We denote with *Σ*^+^ the pseudo-inverse for any matrix *Σ*. We use the same condition number from individual-level MultiXcan ($\frac{\lambda_{\text{max}}}{\lambda_{i}}<30$) as threshold. For S-MultiXcan-significant associations across 100 public traits, we found a median number of available models of 9 (*IQR* = 10), with ∼ 80% of components surviving the SVD threshold.

#### Estimating significance

To quantify significance of the inferred multi-tissue gene-level association, we use the fact that the regression coefficient estimates follow (approximately) a multivariate normal distribution: $\hat{g}\sim N(g,\sigma_{e}^{2}{\left(T^{t}T\right)}^{-1})$. Under the null hypothesis of no association, it follows that ${\hat{g}}^{t}\frac{T^{t}T}{\sigma_{e}^{2}}\hat{g}\sim \chi_{p}^{2}$ We can then replace $\hat{g}$ with its estimate from the marginal regression:

$$\begin{array}{cc} \frac{{\hat{g}}^{t}\left(T^{t}T\right)\hat{g}}{\sigma_{e}^{2}} & =\frac{{\hat{\gamma }}^{t}D{\left(T^{t}T\right)}^{-1}T^{t}T{\left(T^{t}T\right)}^{-1}D\hat{\gamma }}{\sigma_{e}^{2}}\\ & =\frac{{\hat{\gamma }}^{t}D}{\sigma_{e}}{\left(T^{t}T\right)}^{-1}\frac{D\hat{\gamma }}{\sigma_{e}}\\ & \approx \frac{{\hat{\gamma }}^{t}1\left(n-1\right)}{\sigma_{\epsilon }}{\left(T^{t}T\right)}^{-1}\frac{\left(n-1\right)1\hat{\gamma }}{\sigma_{\epsilon }}\\ & \approx {\hat{\gamma }}^{t}\frac{\sqrt{n-1}}{\sigma_{\epsilon }}{\left(\frac{T^{t}T}{n-1}\right)}^{-1}\frac{\sqrt{n-1}}{\sigma_{\epsilon }}\hat{\gamma }\\ & \approx {\hat{z}}^{t}Cor{\left(T\right)}^{-1}\hat{z} \end{array}$$

where *Cor*(**T**) is the autocorrelation of **T**, and $\hat{z}$ is the *p*-vector of marginal analysis z-scores, *γ*_*j*_/*se*(*γ*_*j*_). We have used $\sigma_{e}^{2}\approx \sigma_{\epsilon }^{2}$ as an approximation (i.e. the residual variance of the *marginal* regression as approximation of the residual variance of the *joint* regression). This simplification is conservative, and based on our comparison to the individual multivariate results we consider the loss of efficiency acceptable.

In practice, we will use the SVD pseudo-inverse *Cor*(**T**)^+^ as explained in the previous section, and a *χ*^2^-test: ${\hat{z}}^{t}Cor{\left(T\right)}^{+}\hat{z}\sim \chi_{k}^{2}$, with *k* the number of components surviving the SVD pseudoinverse.

#### Application to GWAS summary statistics

109 public GWAS and GWAS meta-analysis summary statistics data sets were downloaded and analyzed with S-PrediXcan and S-MultiXcan, using the 44 prediction models from GTEx tissues in release version 6p. The list of traits and their Consortium/publication information is available in S4 Dataset.

A type 1 Diabetes study from the Wellcome Trust Case-Control Consortium [36] was acquired from WTCCC (https://www.wtccc.org.uk/). The individual-level data was analyzed with MultiXcan; and GWAS summary statistics were obtained using PLINK2 [37], to enable computation of S-PrediXcan and S-MultiXcan.

### Implementation and computation

Prediction Models were obtained from http://predictdb.org/ resource. These models were trained using Elastic Net as implemented in R’s package *glmnet* [38], with a mixing parameter *α* = 0.5, on 44 tissue studies from GTEx’ release version 6p. The underlying GTEx study data was obtained from dbGaP with accesion number phs000424.v6.p1. Please see [10] for details. We implemented MultiXcan and S-MultiXcan using python scientific packages, working up from existing software in the MetaXcan package. S-PrediXcan, PrediXcan, MultiXcan and S-MultiXcan analysis were computed using the Center for Research Informatics’ high performance cluster at the University of Chicago. PrediXcan, S-PrediXcan, MultiXcan and S-MultiXcan results have been uploaded to the http://gene2pheno.org resources. The databases are open to the research community for arbitrary programmatic query.

## Supporting information

S1 Supplementary Note Simulation description. (PDF)

S1 Dataset Summary statistics for 222 UK Biobank traits used in the MultiXcan analysis included in S1_datatxt. Columns are: **tag**: trait, gene2pheno.org display name; **n_predixcan_significant**: Number of Bonferroni-significant PrediXcan results; **n_multixcan_significant** number of Bonferroni-significant results for MultiXcan; **n_predixcan_only** number of results only significant in PrediXcan; **n_multixcan_only** number of results only significant in MultiXcan.
(TXT)

S2 Dataset Significant associations for MultiXcan on UK Biobank included in S2_data.txt. Columns are: **phenotype**: trait, gene2pheno.org display name; **gene**: Ensembl id; **gene_name**: HUGO name; **pvalue**: p-value of the S-MultiXcan association; **n_models** number of prediction models available for the gene; **n_used** number of independent components surviving PCA selection; **n_samples**: number of individuals available.
(TXT)

S3 Dataset Significant associations for PrediXcan on UK Biobank included in S3_data.txt. Columns are: **Phenotype**: trait, gene2pheno.org display name; **model**: GTEx tissue where the model was trained; **gene**: Ensembl Id; **gene_name**: HUGO name; **zscore** PrediXcan association Z-score, **pvalue** PrediXcan association p-value; **n_samples**: number of individuals available.
(TXT)

S4 Dataset List of Genome-wide Association Meta Analysis (GWAMA) Consortia and phenotypes included in S4_data.txt. Columns are consortium name, study name, gene2pheno.org display name, study sample size, study population, URL of portal where data was downloaded from, link to pubmed entry if available.
(TXT)

S5 Dataset Summary statistics for 109 traits used in the MultiXcan analysis included in S5_data.txt. Columns are: **tag**: gene2pheno.org display name; **consortium**: Consortium Name; **name**: study name; **n_spredixcan_significant**: Number of Bonferroni-significant S-PrediXcan results; **n_smultixcan_significant** number of Bonferroni-significant results for MultiXcan; **n_spredixcan_only** number of results only significant in S-PrediXcan; **n_smultixcan_only** number of results only significant in S-MultiXcan.
(TXT)

S6 Dataset Significant associations for Summary-MultiXcan on public GWAS included in S6_data.txt. Columns are: **tag**: gene2pheno.org display name; **consortium**: Consortium Name; **name**: study name; **gene**: Ensembl id; **gene_name**: HUGO name; **pvalue**: p-value of the S-MultiXcan association; **n** number of S-PrediXcan results available for the gene; **n_indep** number of independent components surviving SVD; **p_i_best** best p-value of S-PrediXcan;**t_i_best** tissue that presented best S-PrediXcan result; **p_i_worst** worst p-value of S-PrediXcan; **t_i_worst** tissue that presented worst S-PrediXcan result.
(TXT)

S7 Dataset Significant associations for Summary-PrediXcan on public GWAS. Significant results included in **S7_data.txt**. Columns are: **consortium**: Consortium Name; **name**: study name; **tag**: gene2pheno.org display name; **gene**: Ensembl Id; **gene_name**: HUGO name; **model** GTEx tissue where model was trained; **zscore** S-PrediXcan association Z-score, **pvalue** S-PrediXcan association p-value.
(TXT)

S8 Dataset MultiXcan-significant associations with modest individual model significance from UK Biobank traits included in S8_data.txt. Columns are: **trait**: UK Biobank trait name and code; **n_flagged**: number of significant genes with best individual model p-value >10^−4^; **n_significant**: number of Bonferroni-significant genes; **percent**: percentage of **n_flagged** to **n_significant**. MultiXcan significance was computed with condition number 30 and the individual model effects’ significance obtained from PrediXcan.
(TXT)

S9 Dataset MultiXcan and S-MultiXcan associations for WTCCC Type 1 Diabetes study included in S9_data.txt. Columns are: **gene**: gene’s ensemble id; **pvalue**: significance achieved;**method**: a label specifying that either MulTiXcan, S-MulTiXcan with naive covariance from predicted expression, or S-MulTiXcan with correction for missing SNPs was ran.
(TXT)

S1 Fig Predicted expression correlation for gene *SLC5A6*. We observe a high degree of predicted expression correlation, in agreement with recent publications on the high degree of mechanism sharing across tissues [9]. This behavior is exhibited in most genes.
(TIF)

S2 Fig Summary-MultiXcan vs MultiXcan for miscellaneous traits. There is a satisfactory agreement between the individual-level and the summary-level versions of MultiXcan in UK Biobank traits.
(TIF)

S3 Fig Distribution of MultiXcan significance under the null hypothesis of no association. Here we use a simulated trait, generated from a standard normal distribution as the phenotype. We perform MultiXcan, regressing the simulated phenotype on predicted expression for 17,435 genes in 1,000 individuals from the UK Biobank. As described in the Methods, we drop principal components of small variation to avoid multi collinearity. We keep the number of principal components so that the condition number of the covariance matrix of the predicted expression across tissues (ratio of the maximum and minimum eigenvalues) is below 30.**Panel a** compares the MultiXcan p-values to the expected uniform distribution. Most points (genes) lie on the identity line showing no obvious inflation or deflation.**Panel b** compares the distribution of p-values with and without regularization.
(TIF)

S4 Fig MultiXcan simulations for different synthetic traits. For each gene, we simulate traits as different combinations of predicted expression from multiple tissues in one thousand individuals from the UK Biobank. We add a noise term from the normal distribution with variance chosen so that 1% of the total variance in the trait is explained by predicted expression. For each trait, we show results from running MultiXcan with no regularization, MultiXcan with regularization (condition number < 30), PrediXcan with ‘best’ single tissue (either the single causal tissue or most significant p-value in each gene). For a trait with specific causal tissues, we also show MultiXcan using only them.**Panel a** compares p-value distributions for traits generated from a single tissue (Whole Blood, 6588 genes available). In this case, PrediXcan using whole blood prediction outperforms MultiXcan as expected from the fact that MultiXcan’s statistic becomes less significant when more explanatory variables of no effect are used; both unregularized and PCA-regularized MulTiXcan are similarly affected.**Panel b** Uses a trait built from the combination of five brain tissues (Cerebellum, Cerebellar Hemisphere, Hippocampus, Cortex, Frontal Cortex BA9, 488 genes in the intersection of tissue models). As expected, MultiXcan using only the causal tissues performs best. MultiXcan using all tissues displays the second best performance, with the regularized version being slightly better than the unregularized version. PrediXcan (i.e. a single tissue) has the lowest performance.**Panel c** shows simulations when all tissues are causal (for 1000 random genes); MultiXcan with PCA regularization has slightly better performance than unregularized MultiXcan, and ‘best tissue’ PrediXcan has a significantly lower performance.
(TIF)

S5 Fig Trend in MultiXcan significance for increasing number of included tissues. For each gene, we simulate traits as different combinations of predicted expression from multiple tissues in one thousand individuals from the UK Biobank. We add a noise term from the normal distribution with variance chosen so that 1% of the total variance in the trait is explained by predicted expression. The top panel shows traits generated from the combination of 5 brain tissues (Cerebellum, Cerebellar Hemisphere, Hippocampus, Cortex, Frontal Cortex BA9; top panel), and the bottom panel a combination of all available tissues. These traits were analyzed through MultiXcan both with PCA regularization and without regularization. The lines correspond to smoothed conditional means, and the gray area displays the confidence intervals. We observe that PCA regularization has increased power over no regularization with larger effect as the number of included tissues increases. When the number of causal tissues is small (“5 Brains”), significance decreases when more tissue models are available, and the regularized and unregularized MultiXcan perform similarly. This is expected since extra uninformative components add noise and reduce power. Conversely, when all tissues are causal, significance increases as we increase the number of included tissues. Regularized MultiXcan achieves higher significance than unregularized MultiXcan.
(TIF)

S6 Fig Stability of significance for different condition number thresholds in the PCA regularization. Using simulated traits in two scenarios (5 brain causal tissues and all causal tissues, as described in the Supplementary Note), we display MultiXcan’s significance distribution for different PCA regularization thresholds. In both scenarios the significance remains relatively constant for all thresholds tested. More stringent regularization thresholds achieve slightly higher significance. We consider the threshold of 30 to be a conservative choice.
(TIF)

S7 Fig Association detection for PrediXcan and MultiXcan using FDR. The number of FDR-significant associations are shown for PrediXcan using both a single tissue and all tissues, and MultiXcan. Using *FDR* < 0.05, we observe that the number of significant associations for both PrediXcan and MultiXcan increase significantly, and their difference decreases. Using smaller FDR thresholds increases the difference, and for *FDR* < 10^−4^ we observe a similar number of detections as when performing traditional multiple-testing correction at 0.05/*n*. This is consistent with Bonferroni correction being overly conservative because the hypotheses are not independent.
(TIF)

S8 Fig Accuracy of predicted expression inference. A scatter plot of association significance between MultiXcan and S-MultiXcan is shown for the Wellcome Trust Case-Control Type 1 Diabetes study. The left plot uses the covariance matrix computed from predicted expression in a reference panel (GTEx). The right plot uses predicted expression covariance taking into account missing SNPs (i.e.: using only SNPs in the intersection between reference panel and the GWAS study). We observe that using expression predicted in the reference panel without correction leads to false positives and negatives, as the inferred covariance is inaccurate.
(TIF)

S1 Table Summary statistics comparing S-MultiXcan and S-PrediXcan on public GWAS. (PDF)
