# Supplementary material for: Integrating predicted transcriptome from multiple tissues improves association detection
Source: PLoS Genet. 2019 Jan 22;15(1):e1007889. doi: 10.1371/journal.pgen.1007889 (PMC6358100; doi:10.1371/journal.pgen.1007889)
Supplement: S1 Supplementary Note — (PDF) [file pgen.1007889.s001.pdf]

Supplementary Note: Simulations

In order to compare MultiXcan and PrediXcan under different controlled scenarios, we performed a wide variety of simulations using real genotype data. First, a thousand individuals from the UK Biobank cohort were chosen at random. Then, we predicted their gene expression using the models trained on 44 tissues from GTEx data. We then generated different sets of traits for each gene, and ran PrediXcan, MultiXcan without regularization, and MultiXcan with PCA regularization.

We first generated a random trait drawing values from a standard normal distribution, $N(0,1)$, for every individual in the sample. We compare the distribution of p-values to the expected uniform distribution in S3 Fig-a and observe proper calibration. In this case, we observe no significant difference between MultiXcan with and without PCA regularization in S3 Fig-b.

We also generated traits from linear combinations of predicted expression in real individuals, for each gene, with an additional noise term drawn from a Gaussian distribution with a scale chosen to account for 99% of the expected variance. We compared significance between PrediXcan and MultiXcan (both with and without PCA regularization) in S4 Fig for different simulation scenarios.

In the first simulation case, shown in S4 Fig-a, traits were simulated from predicted expression for a single tissue: $\mathbf{y}_{\text{gene j}} = \mathbf{t}_{\text{whole blood, gene j}} + \epsilon$. Here, $\mathbf{y}_{\text{gene j}}$ is the simulated trait for gene j; $\mathbf{t}_{\text{whole blood, gene j}}$ predicted expression in Whole Blood tissue for gene j, and ϵ is a random Gaussian noise term with a scale such that it accounts for 99% of the expected variance (i.e. $\sqrt{99}N(0,1)$).

Another simulation case uses linear combinations of 5 brain tissues (Cerebellum, Cerebellar Hemisphere, Hippocampus, Cortex, Frontal Cortex BA9), shown in S4 Fig-b, defined from $\mathbf{y}_{\text{gene j}} = \sum_{i=1}^{5} \mathbf{t}_{\text{Brain i, gene j}} + \epsilon$ with $\mathbf{t}_{\text{Brain i, gene j}}$ expression in tissue i among the chosen brain panels. The last simulation case uses all available tissues for a thousand genes, shown in S4 Fig-c. In both cases, the noise term was chosen for each gene so that it accounts for 99% of the expected variance.

MultiXcan outperforms PrediXcan in our simulations except for the trivial case with only a single causal tissue (S4 Fig-a). This is caused by MultiXcan's significance being penalized when additional explanatory variables that have no effect are used. In a real application, the true "causal" tissues are not generally known; thus we recommend using MultiXcan over PrediXcan in the general case.

In the case with a few causal tissues (S4 Fig-b), PrediXcan is barely able to detect any significant association. As expected, the highest significance is achieved when using MultiXcan with only the causal tissues. PCA regularization increases significance as it discards less informative explanatory variables. We observed a similar behavior in traits generated from all available tissues (S4 Fig-c). Although more modest, PCA regularization still displays an increase in association significance because less informative components are discarded.

Based on these simulations, we conclude that MultiXcan is more convenient

than PrediXcan in the general case where multiple tissues are expected to be involved in a trait. Restricting to causal tissues when known (i.e. a hypothetical psychiatric disease depending only on brain tissues) can improve power. Since PCA regularization also fixes the numerical instability caused when predicted expression from multiple tissues are highly correlated, we highly recommend running MultiXcan with it. This becomes more important when more tissues are available, as can be seen in S5 Fig.

We ran MulTixcan using different condition numbers to assess the stability of the regularization, and display the results in S6 Fig. Significance decreases with higher condition numbers, since a larger number of uninformative components are used, decreasing significance of the overall fit. Significance levels display little change between condition number 30 and 100. Based on this observation, we choose 30 as a conservative threshold for our analysis. There is a degree of arbitrariness to this threshold as the true number of causal tissues and their correlation is not known in most cases; we suggest users to run MultiXcan with different condition numbers to verify its stability in each case.
