# Supplementary figures and images for: Integrating predicted transcriptome from multiple tissues improves association detection
Source: PLoS Genet. 2019 Jan 22;15(1):e1007889. doi: 10.1371/journal.pgen.1007889 (PMC6358100; doi:10.1371/journal.pgen.1007889)

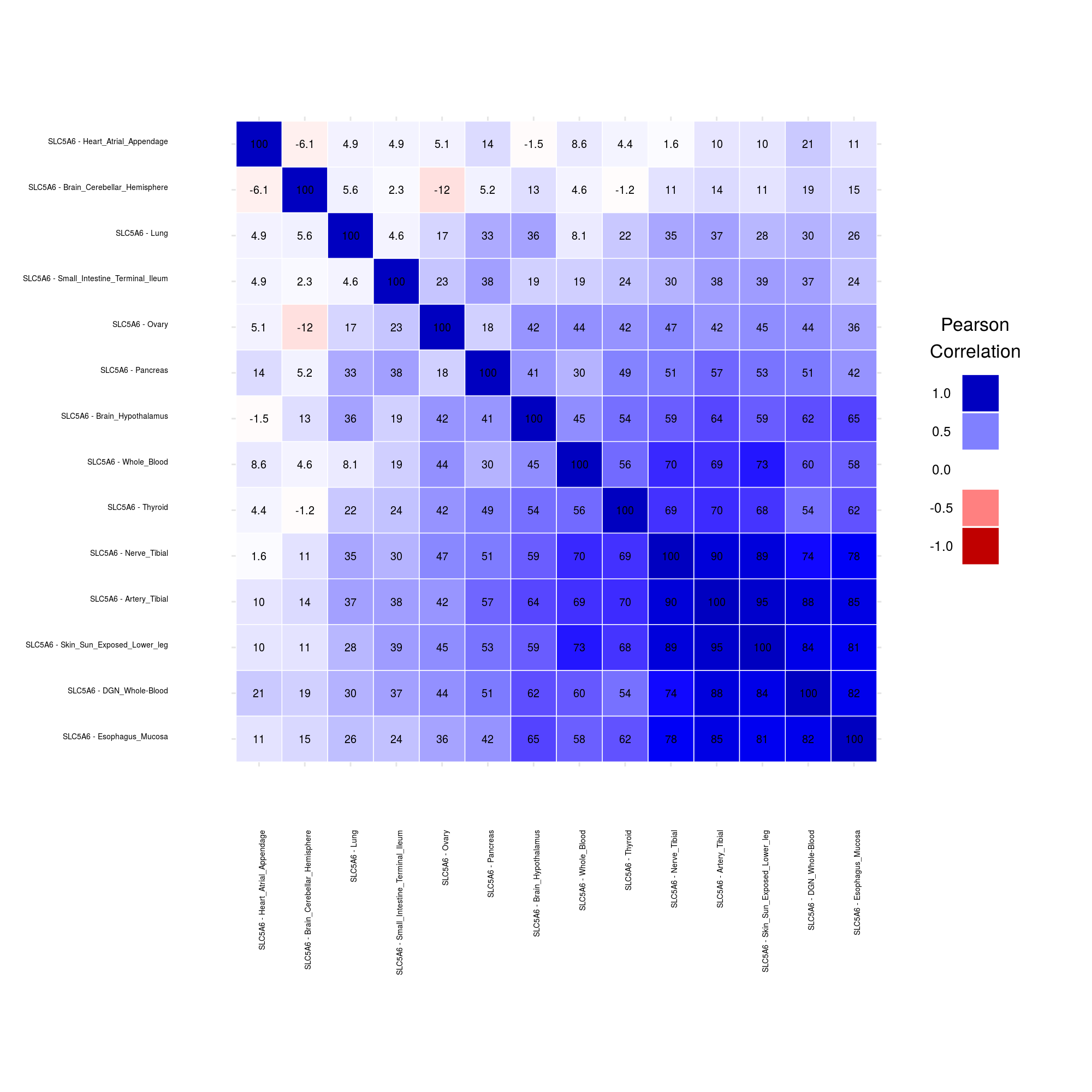

Supplement: S1 Fig — We observe a high degree of predicted expression correlation, in agreement with recent publications on the high degree of mechanism sharing across tissues [9]. This behavior is exhibited in most genes. (TIF) [file pgen.1007889.s011.tif]

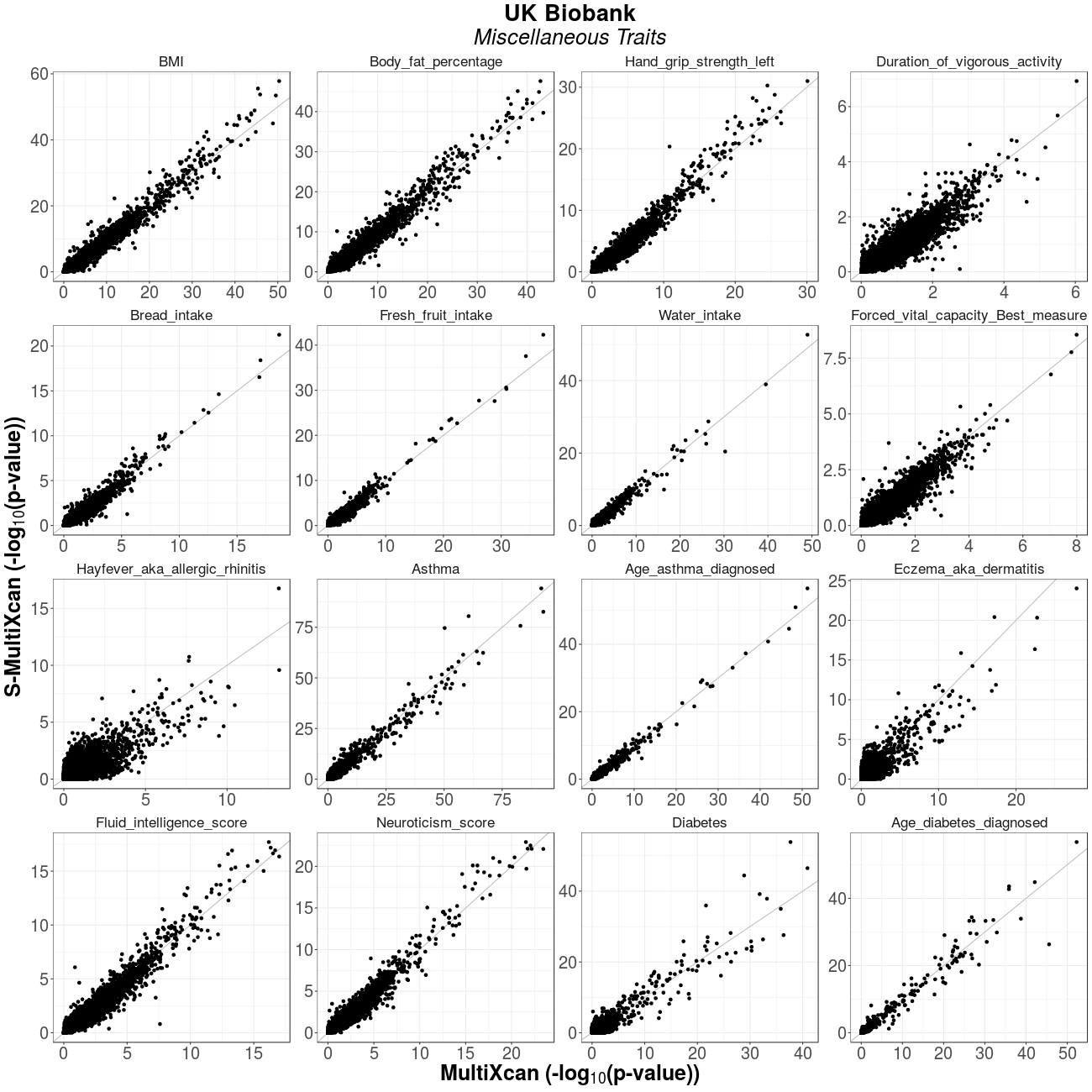

Supplement: S2 Fig — There is a satisfactory agreement between the individual-level and the summary-level versions of MultiXcan in UK Biobank traits. (TIF) [file pgen.1007889.s012.tif]

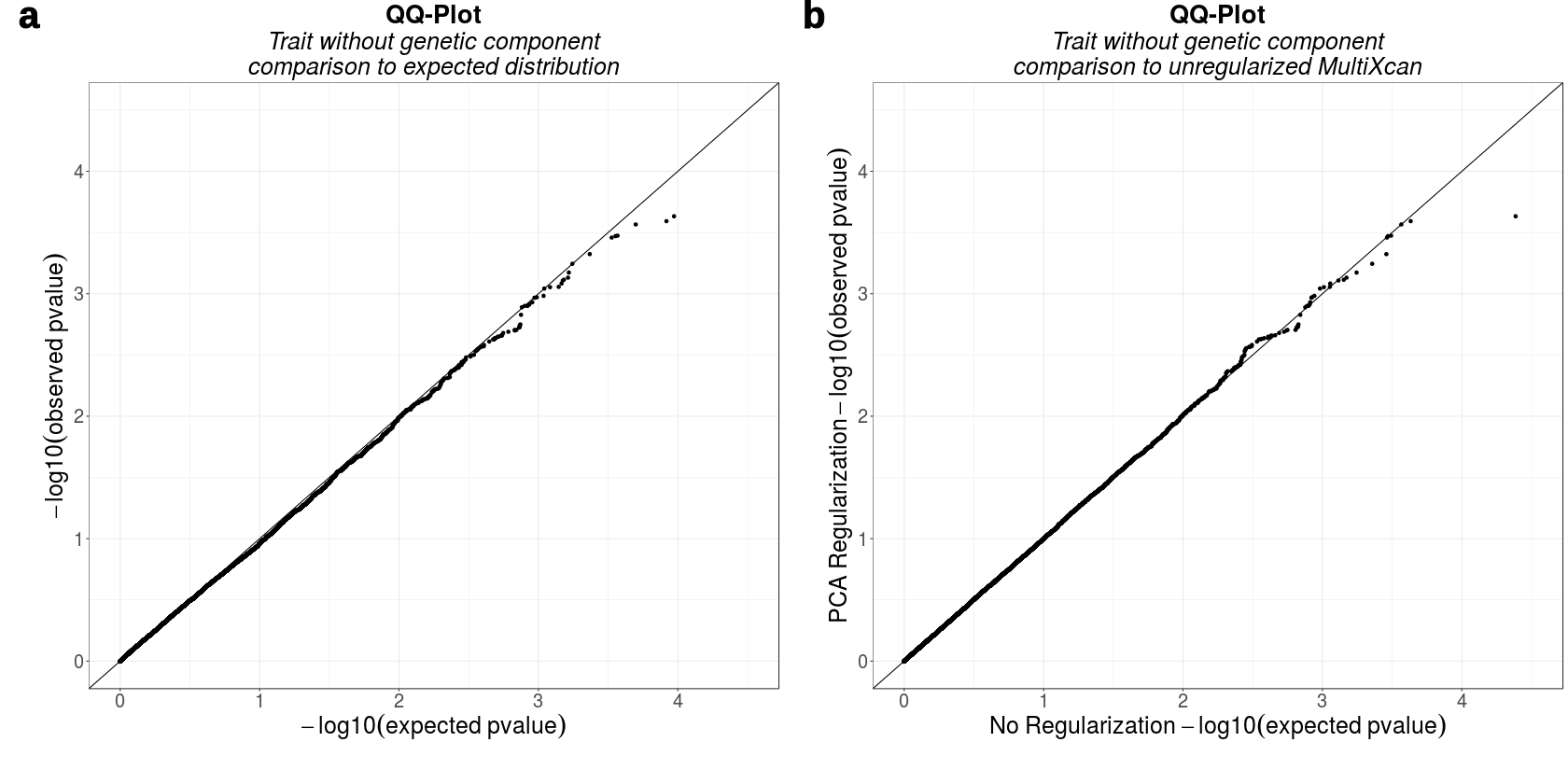

Supplement: S3 Fig — Here we use a simulated trait, generated from a standard normal distribution as the phenotype. We perform MultiXcan, regressing the simulated phenotype on predicted expression for 17,435 genes in 1,000 individuals from the UK Biobank. As described in the Methods, we drop principal components of small variation to avoid multi collinearity. We keep the number of principal components so that the condition number of the covariance matrix of the predicted expression across tissues (ratio of the maximum and minimum eigenvalues) is below 30.Panel a compares the MultiXcan p-values to the expected uniform distribution. Most points (genes) lie on the identity line showing no obvious inflation or deflation.Panel b compares the distribution of p-values with and without regularization. (TIF) [file pgen.1007889.s013.tif]

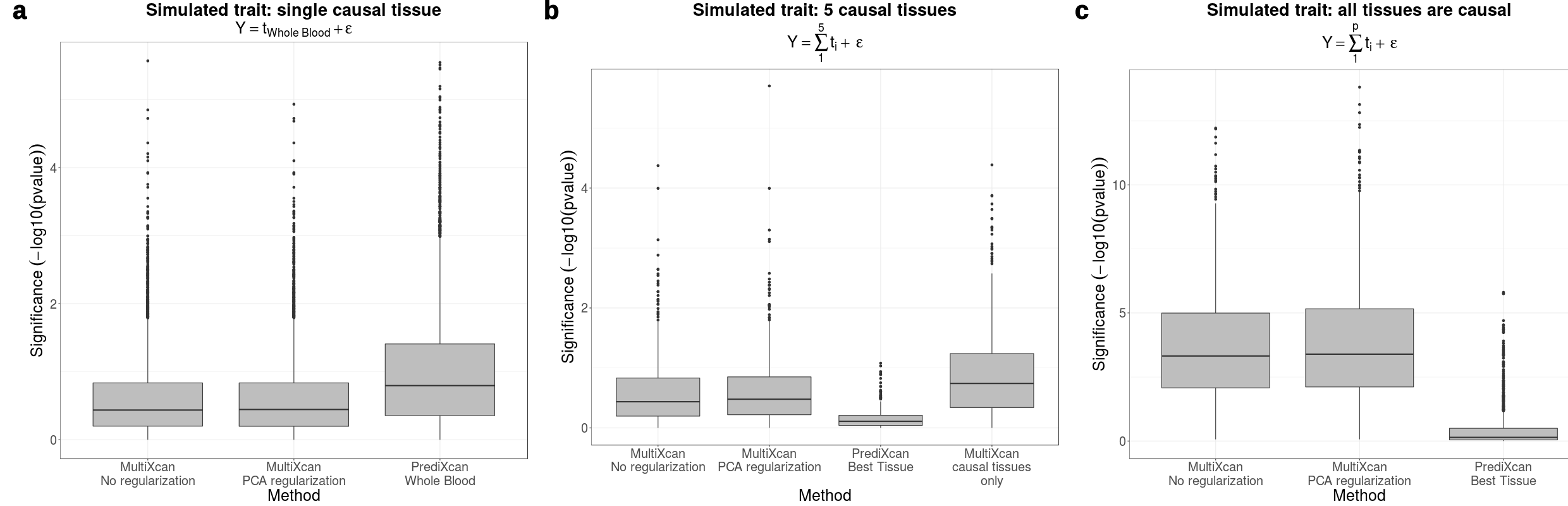

Supplement: S4 Fig — For each gene, we simulate traits as different combinations of predicted expression from multiple tissues in one thousand individuals from the UK Biobank. We add a noise term from the normal distribution with variance chosen so that 1% of the total variance in the trait is explained by predicted expression. For each trait, we show results from running MultiXcan with no regularization, MultiXcan with regularization (condition number < 30), PrediXcan with ‘best’ single tissue (either the single causal tissue or most significant p-value in each gene). For a trait with specific causal tissues, we also show MultiXcan using only them.Panel a compares p-value distributions for traits generated from a single tissue (Whole Blood, 6588 genes available). In this case, PrediXcan using whole blood prediction outperforms MultiXcan as expected from the fact that MultiXcan’s statistic becomes less significant when more explanatory variables of no effect are used; both unregularized and PCA-regularized MulTiXcan are similarly affected.Panel b Uses a trait built from the combination of five brain tissues (Cerebellum, Cerebellar Hemisphere, Hippocampus, Cortex, Frontal Cortex BA9, 488 genes in the intersection of tissue models). As expected, MultiXcan using only the causal tissues performs best. MultiXcan using all tissues displays the second best performance, with the regularized version being slightly better than the unregularized version. PrediXcan (i.e. a single tissue) has the lowest performance.Panel c shows simulations when all tissues are causal (for 1000 random genes); MultiXcan with PCA regularization has slightly better performance than unregularized MultiXcan, and ‘best tissue’ PrediXcan has a significantly lower performance. (TIF) [file pgen.1007889.s014.tif]

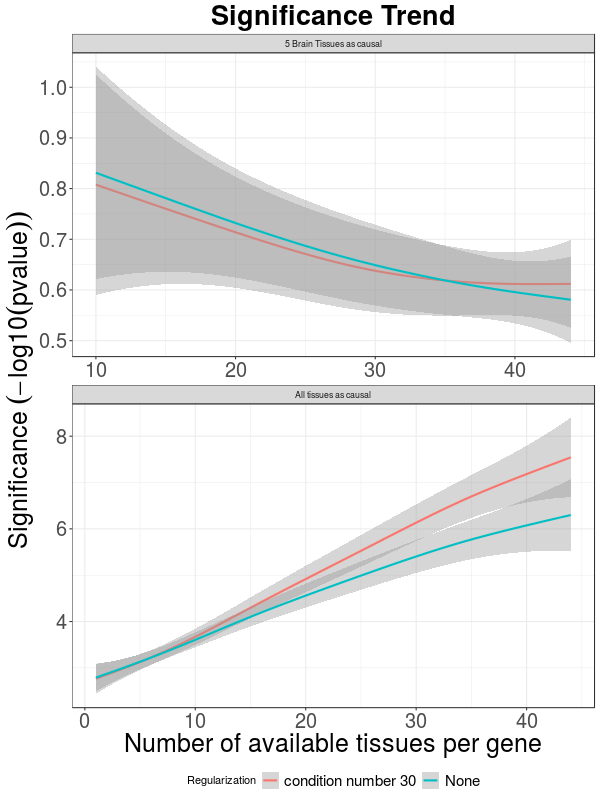

Supplement: S5 Fig — For each gene, we simulate traits as different combinations of predicted expression from multiple tissues in one thousand individuals from the UK Biobank. We add a noise term from the normal distribution with variance chosen so that 1% of the total variance in the trait is explained by predicted expression. The top panel shows traits generated from the combination of 5 brain tissues (Cerebellum, Cerebellar Hemisphere, Hippocampus, Cortex, Frontal Cortex BA9; top panel), and the bottom panel a combination of all available tissues. These traits were analyzed through MultiXcan both with PCA regularization and without regularization. The lines correspond to smoothed conditional means, and the gray area displays the confidence intervals. We observe that PCA regularization has increased power over no regularization with larger effect as the number of included tissues increases. When the number of causal tissues is small (“5 Brains”), significance decreases when more tissue models are available, and the regularized and unregularized MultiXcan perform similarly. This is expected since extra uninformative components add noise and reduce power. Conversely, when all tissues are causal, significance increases as we increase the number of included tissues. Regularized MultiXcan achieves higher significance than unregularized MultiXcan. (TIF) [file pgen.1007889.s015.tif]

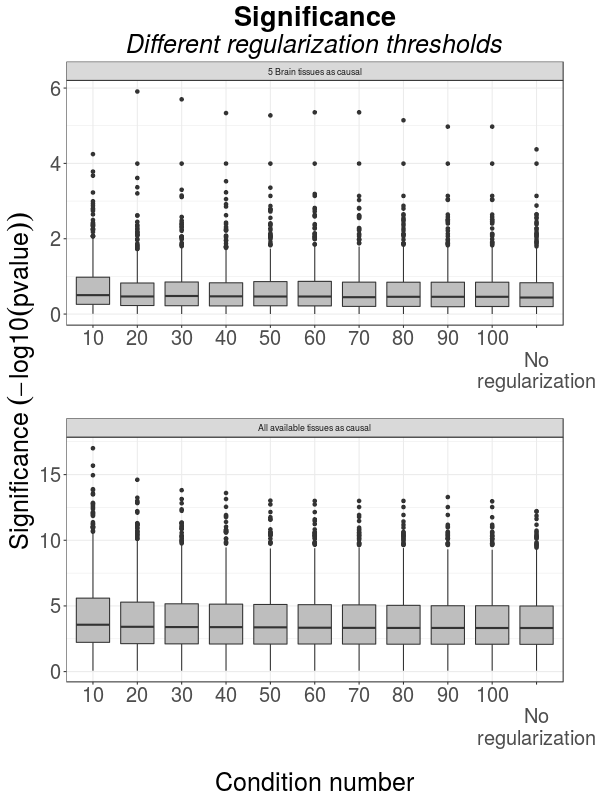

Supplement: S6 Fig — Using simulated traits in two scenarios (5 brain causal tissues and all causal tissues, as described in the Supplementary Note), we display MultiXcan’s significance distribution for different PCA regularization thresholds. In both scenarios the significance remains relatively constant for all thresholds tested. More stringent regularization thresholds achieve slightly higher significance. We consider the threshold of 30 to be a conservative choice. (TIF) [file pgen.1007889.s016.tif]

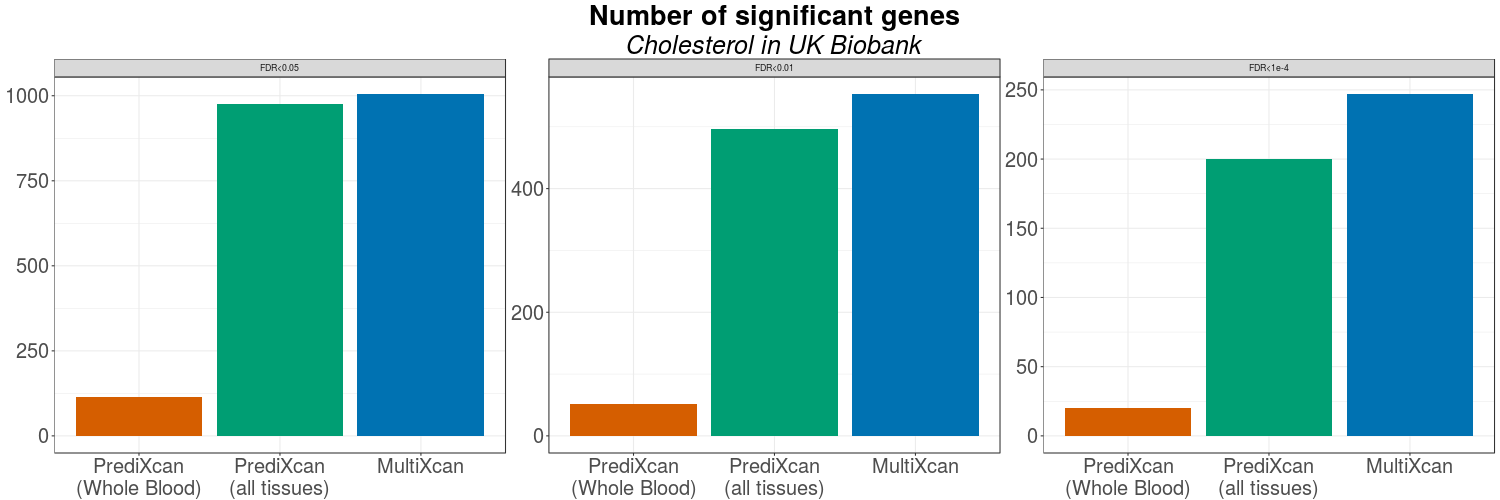

Supplement: S7 Fig — The number of FDR-significant associations are shown for PrediXcan using both a single tissue and all tissues, and MultiXcan. Using FDR < 0.05, we observe that the number of significant associations for both PrediXcan and MultiXcan increase significantly, and their difference decreases. Using smaller FDR thresholds increases the difference, and for FDR < 10−4 we observe a similar number of detections as when performing traditional multiple-testing correction at 0.05/n. This is consistent with Bonferroni correction being overly conservative because the hypotheses are not independent. (TIF) [file pgen.1007889.s017.tif]

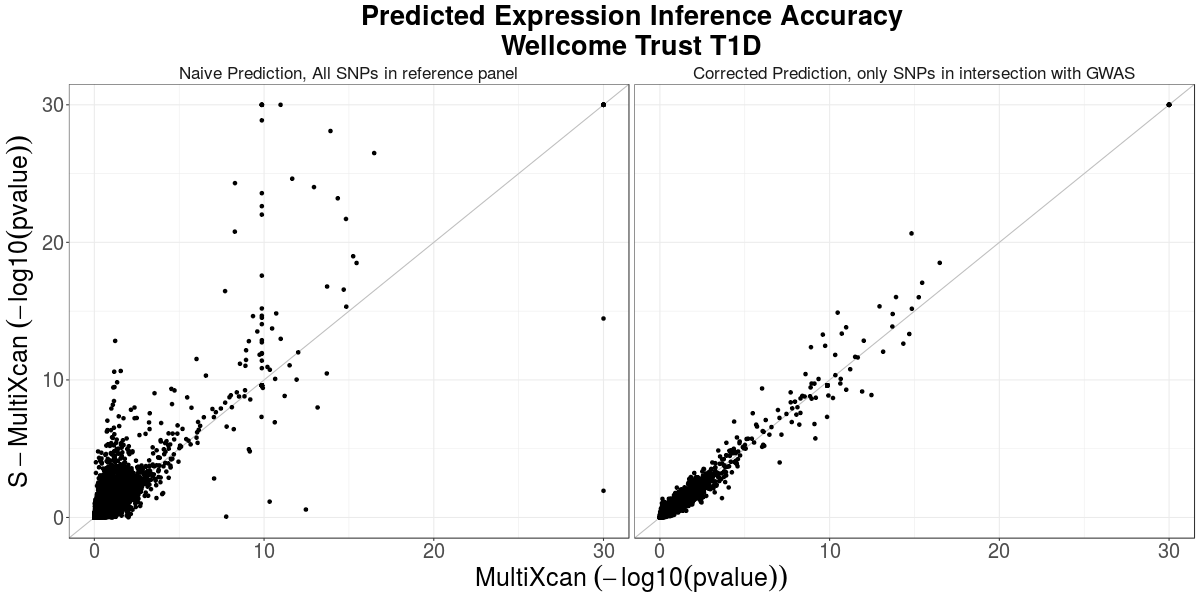

Supplement: S8 Fig — A scatter plot of association significance between MultiXcan and S-MultiXcan is shown for the Wellcome Trust Case-Control Type 1 Diabetes study. The left plot uses the covariance matrix computed from predicted expression in a reference panel (GTEx). The right plot uses predicted expression covariance taking into account missing SNPs (i.e.: using only SNPs in the intersection between reference panel and the GWAS study). We observe that using expression predicted in the reference panel without correction leads to false positives and negatives, as the inferred covariance is inaccurate. (TIF) [file pgen.1007889.s018.tif]
