# Supplementary material for: Integrating predicted transcriptome from multiple tissues improves association detection
Source: PLoS Genet. 2019 Jan 22;15(1):e1007889. doi: 10.1371/journal.pgen.1007889 (PMC6358100; doi:10.1371/journal.pgen.1007889)
Supplement: S1 Table — (PDF) [file pgen.1007889.s019.pdf]

Supplementary Table 1: Summary statistics comparing S-MultiXcan and S-PrediXcan on public GWAS

Traits with more S-MultiXcan-significant associations	51
Traits with more S-PrediXcan-significant associations	20
Tied traits	12
Traits without significant associations	26
Average increase in significant associations for S-MultiXcan *	10.8
Average significant association overlap **	53 %

$*$: average performed across traits where there is at least one S-PrediXcan- or S-MultiXcan-significant association.
$**$: computed as $\frac{\#\text{shared}}{\#union(SM,SP)}$, with SM the S-MultiXcan-significant associations, SP the S-PrediXcan-significant associations, and #shared the number of shared associations.
